# Traditional practices versus modern healthcare: Determinants of traditional medicine use after potential dog bites among dog-owning households in Nigeria

**DOI:** 10.1371/journal.pntd.0012910

**Published:** 2025-03-17

**Authors:** Philip P. Mshelbwala, Kinley Wangdi, Jibrin Idris, Mohammad Mahmudul Hassan, Andrew M. Adamu, Charles E. Rupprecht, Nicholas J. Clark

**Affiliations:** 1 Faculty of Veterinary Medicine, University of Abuja, Abuja, Nigeria; 2 NSW Department of Primary Industries, Orange, New South Wales, Australia,; 3 HEAL Global Research Centre, Health Research Institute, Faculty of Health, University of Canberra, Bruce, Australia; 4 National Centre for Epidemiology and Population Health (NCEPH), Australian National University, Acton, Australia; 5 African Field Epidemiology Network, Abuja, Nigeria; 6 School of Veterinary Science, The University of Queensland, Gatton, Queensland, Australia; 7 Australian Institute of Tropical Health and Medicine, James Cook University, Townsville, Queensland, Australia; 8 College of Forestry, Wildlife & Environment, College of Veterinary Medicine, Auburn University, Auburn, Alabama, United States of America; Swiss Tropical and Public Health Institute: Schweizerisches Tropen- und Public Health-Institut, SWITZERLAND

## Abstract

Canine rabies is endemic in Nigeria, with a low dog vaccination rate. Often, dog bite victims resort to traditional remedies, which can lead to fatalities. Our study investigated factors influencing decisions to seek traditional remedies in Nigeria. We conducted a cross-sectional study in 2022 involving 4,162 dog-owning households. A joint random effect Bayesian regression model was developed to examine the role of sociodemographic, socioeconomic, and infrastructural covariates. This model included a latent variable measuring a respondent’s understanding of rabies risk based on literacy levels and responses to questions about rabies epidemiology. Our results indicated that 27% (95% Confidence Interval [Cl); 26-27) of respondents would preferably seek traditional remedies following a dog bite. Male respondents were 24% more likely than female respondents to seek traditional remedies (odds ratio [OR]: 1.24; 95%, Credible Interval CrI): 1.07-1.31). Similarly, individuals residing in rural areas reported 55% higher likelihood of using traditional remedies than those in urban areas (OR: 1.55; 95% CrI: 1.43–1.67). Respondents residing in areas with no veterinary services reported 35% higher likelihood of using traditional remedies than those near such facilities (OR: 1.35; 95% CrI: 1.15–1.42). Children under 16 years reported 27% lower likelihood of using traditional remedies than adults (OR: 0.73; 95% CrI: 0.49–0.84). Private or unemployed individuals were more likely to seek traditional remedies than civil servants (OR: 1.99; 95% Crl: 1.53-2.37). Respondents with tertiary education reported 42% lower likelihood of using traditional remedies than those without formal education (OR: 0.58; 95% CrI: 0.49–0.62). Our latent variable representing understanding of rabies risk was negatively associated with the probability of seeking traditional remedies (OR: 0.67; 95% CrI: 0.54–0.73). Lastly, poverty was negatively associated with the likelihood of seeking traditional remedies (OR: 0.78; 95% CrI: 0.66–0.82). Our findings provide important insights into healthcare behaviour decisions and their possible associations with rabies outcomes in Nigeria. These results highlight the need to improve public education, enhance access to medical care, and involve traditional healers in rabies prevention and control programs.

## Author summary

Canine rabies is endemic in Nigeria, with low canine vaccination rates. Dog bite victims frequently resort to traditional remedies, which can lead to fatalities. We conducted a study across three states in Nigeria and the Federal Capital Territory to understand the factors influencing the decision to seek traditional therapy. We found that being male, residing in a rural area, and having limited access to veterinary services were significantly associated with the probability of seeking traditional remedies. Children under 17 years of age, respondents with higher levels of education, and civil servants were less likely to use traditional remedies after being bitten by a dog. Also, poverty and a latent understanding of the risks of rabies were negatively associated with the probability of seeking traditional remedies. Our findings highlight the need to improve public education, enhance access to medical care, and involve traditional healers as rabies surveillance informants in rabies prevention and control programs.

## Introduction

Rabies is a significant public health challenge in over 150 countries, predominantly in Asia and Africa [1, 2]. This zoonosis is transmitted primarily by the bite of an infected dog [3]. Despite the availability of effective prophylaxis, rabies causes tens of thousands of human deaths annually, especially in lower- and middle-income countries (LMIC) with limited access to healthcare [2]. Contemporary postexposure prophylaxis (PEP), including immediate wound washing with soap and water, intradermal (ID) or intramuscular (IM) vaccination, and administration of rabies immune globulin (RIG) or monoclonal antibodies (MAbs) are recognized for their efficacy in preventing rabies [4]. However, many individuals’ resort to traditional practices following dog bites [5]. These approaches may include herbal remedies, spiritual rituals, or consultations with traditional healers deeply embedded in cultural beliefs and societal norms [5]. Among 105 confirmed human rabies cases reported between 2008 and 2018 in South Africa, approximately one-third did not receive medical intervention, including bite wound care and vaccination [6]. In a survey conducted in Bangladesh, only 25% of participants received PEP, while the majority resorted to traditional approaches [7]. In North India, a study found a correlation between delays in seeking PEP and the use of traditional remedies after dog bites [8]. An investigation conducted in South Africa identified low awareness and poverty as key factors contributing to the failure to seek PEP following a dog bite [6]. Other studies identified various determinants, such as trust in traditional healers, perceived affordability and accessibility of modern healthcare, and cultural norms for seeking traditional medicine [2,9].

The global framework for the elimination of dog-mediated human rabies highlights five critical pillars for rabies control: Sociocultural, Technical, Organizational, Political, and Resource (STOP-R) [10]. The first sociocultural pillar recognises the importance of community engagement and the influence of local cultural contexts on healthcare practices, but is often overlooked in rabies prevention programs [11]. Understanding the sociocultural drivers behind the preference for a traditional approach rather than an allopathic approach after a dog bite is critical to designing more effective, community-centred rabies prevention strategies.

In Nigeria, canine rabies is endemic, with a low canine vaccination rate. Previous studies reported dog bite victims often resorting to traditional remedies after dog bites [12]. This included eating the abdomen of the biting dog, using herbal concoctions, consuming the cooked liver of a stray dog and placing pulled hair from the dog’s neck on the bite wound, with mortalities recorded in several cases [12]. This increased preference for traditional over allopathic medical intervention across Nigeria raises critical concerns about the effectiveness of rabies prevention strategies. The underlying reasons for choosing traditional over allopathic care after a dog bite remain poorly understood, particularly in Nigeria. Unravelling these reasons is crucial for developing improved disease prevention and control strategies. Hypothetical surveys have helped understand healthcare beliefs by eliciting perceptions and intentions based on the Health Belief Model (HBM) [13, 14]. The HBM suggests that perceived susceptibility, severity, benefits, and barriers shape health behaviours. Using hypothetical scenarios, these surveys provide insights into how individuals might respond to real health threats, helping design effective health interventions[15, 16].

We used a joint random effect Bayesian regression model to investigate factors influencing decisions to seek traditional remedies following dog bites in Nigeria. Our objective was to seek insights that would inform more effective rabies prevention and control interventions in Nigeria and other LMICs where cultural and socioeconomic factors shape health-seeking behaviours.

## Materials and methods

### Ethics Statement

The study received approval from the Federal Capital Territory Health Research Ethics Committee (FCT HREC) under the reference/FHREC/2019/01/04/21–01-19. Children who owned dogs completed the survey under the guidance of adults, as children are more predisposed to dog bites and at higher risk of exposure to rabies virus [2]. Formal verbal consent was obtained from the parents or guardians of all child participants. We obtained verbal consent from all participants included in the survey as enshrined in the Helsinki Declaration 2001 [17].

### Study area

The study was conducted between June and December 2022 in four states in Nigeria: Anambra, Enugu, Kaduna, and the Federal Capital Territory (FCT) (S1 Appendix). Our earlier paper detailed the rationale for selecting these locations [18]. Briefly, we selected locations where data would be collected by stratification using the level of state rabies reporting from the national surveillance data and reports of rabies from published studies that suggested more canine rabies in the Northwest and Southeast [19]. We selected Kaduna in the Northwest (Ikara and Sabon Gari LGAs), the FCT in the northcentral (Kuje and Bwari LGAs), and Enugu and Anambra (Idemili South and Ekwusigo) in the Southeast.

### Sociodemographic variables

We conducted a cross-sectional study involving 4,162 dog-owning households across Nigeria. The methods of data collection were described in detail elsewhere [18]. Briefly, we collected information on sociodemographic characteristics of dog owners, dog-keeping practices, access to veterinary care, dog rabies vaccination status, knowledge about rabies, and individual dog information. The questionnaire was administered to an adult household member in English, Hausa, Yoruba, and Igbo. For households with children as dog owners, parents or guardians completed the survey on behalf of the child to ensure understanding and accuracy. Data were collected using Kobo Toolbox, a free and open-source platform for mobile data collection [20]. After completing the data collection, we downloaded the datasets from Kobo Toolbox and performed data cleaning and preprocessing using R statistical software before proceeding with the analysis [21].

### Socioeconomic and infrastructure variables

We wanted to understand the influence of socioeconomic and infrastructure factors on care seeking behaviours after a potential dog bite. Therefore, we obtained additional data from sources other than household data. This included poverty raster maps estimating the proportion of people per grid square living in poverty—defined by the $1.25 and $2 a day thresholds—at approximately 1 km resolution [22]; a literacy raster map showing the proportion of literate men and women aged 15–49 per grid square (at approximately 1 km resolution) [23]; and an urban extent grid (version 1) indicating the proportion of rural and urban areas extracted from the Global Rural-Urban Mapping Project (GRUMP v1) [24]. Data on the distance to road networks provide mean distances in meters from each grid cell [25]. All rasters can be found in the S1 Appendix. All surveyed households were geocoded and overlaid onto these raster datasets using the R programming language [21]. We used the raster package to extract the precise geographic locations of the households [26]. The extracted spatial and primary survey data were compiled and stored in Microsoft Excel [27]. Before conducting statistical analyses, we standardised all continuous variables to unit variance by subtracting their means and dividing by their standard deviations using the scale function in R to ensure comparability across regression coefficients associated with these variables.

## Statistical analysis

### Variable selection

**Fig 1:**
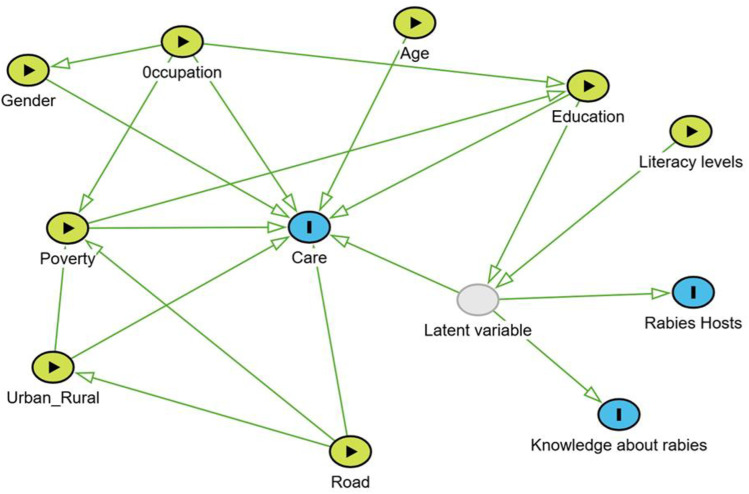
Directed Acyclic Graph (DAG) informing regression models: the graph illustrates postulated causal relationships between the exposure (green) and outcomes (blue). Grey nodes represent latent variables, which reflect the understanding of basic rabies epidemiology, including knowledge about host species and transmission pathways via dog bites.


We constructed a directed acyclic graph (DAG) to represent our assumptions about the relationships between predictor variables and the likelihood of seeking traditional remedies after a dog bite. Previous research and expert opinions informed the DAG [28, 29]. We used the DAG to identify sufficient adjustment sets for each exposure-outcome relationship of interest, applying the do-calculus method with DAGitty software [25]. Our DAG included a latent variable to represent a respondent’s understanding of the risk of rabies, which we assumed was directly related to the respondent’s literacy levels (from the literacy raster) and their responses to questions about rabies epidemiology. Similar approaches have been used to target questions related to a latent understanding of rabies [18,30]. These included a basic understanding of the mode of rabies virus transmission, the knowledge that rabies affects both humans and other animals, and a history of having heard about rabies - which are good indicators for risk assessment for rabies (Fig 1). Our latent variable was assumed to follow a normal distribution. We modelled its mean using a linear predictor to capture the proposed dependency on the respondents’ literacy. The variance parameter (*σ*_*risk*_) was not identifiable at values approaching 0 (which would result in the latent variable perfectly matching the measured literacy variable), so we specified an informative uniform prior density to capture our hypothesis that a person’s understanding of the risk of rabies was strongly, but not solely, correlated with literacy:

$$\begin{array}{l} risk∼\text{Normal }\left(\mu_{risk},\sigma_{risk}\right)\\ \mu_{risk}=\beta_{risk}\cdot \text{literacy}\\ \sigma_{risk}∼\text{Uniform }\left(0.5,1\right)\\ \beta_{risk}∼\text{Normal }\left(0.25,1\right) \end{array}$$

To improve our inference about the latent variable, we used two binary responses—whether respondents had heard of rabies (yes or no) and whether they knew rabies virus is transmitted through dog bites (true or false)—to estimate the latent variable. Both variables were combined into a two-column matrix using the cbind function in R, which we referred to as ‘knowledge.’ We then used the observed vector of binary responses (knowledge) as outcome variables, assumed to be drawn from Bernoulli distributions with unknown parameters (pi). We modelled the *p*_*i*_s as directly depending on the latent variable (risk) and intercept terms (α_*i*_) to capture systematic variation in average responses to each about whether respondents had heard of rabies (yes or no) and whether they knew rabies virus is transmitted through dog bites (true or false)


$$\begin{array}{l} \text{logit }\left(p_{\text{knowledge }[i]}\right)=\alpha_{\text{knowledge }[i]}+risk\\ \alpha_{\text{knowledge}}∼\text{Normal }\left(-0.25,1\right) \end{array}$$

We developed an ordinal regression model to improve our inference about the latent variable (risk). The outcome of interest was an ordered response to a survey question about which host species are susceptible to rabies virus. Respondents who answered correctly (indicating that rabies affects both humans and animals) scored 3, those who identified one correct host scored 2, and those who answered ‘neither humans nor animals’ scored 1.

We used an ordinal regression model with latent variable as a predictor. Ordinal outcomes can be modelled by introducing a latent continuous variable related to the observed outcomes via a categorical observation model with a cumulative logit link function. In this model, a set of internal cutpoints (*c*_*k*_)partitions the log-cumulative odds of the *K* ordered response categories. To ensure a robust prior model, especially when ordinal data are weakly informative in some or all response categories, we used a Dirichlet prior on the internal cutpoints [31].

*For k in 1,2 … K ordered rabies host response categories*

*host *∼ Categorical*(p*_*host*_)


$$\begin{array}{l} p_{host[}1_{]}=q_{1}\\ p_{host[}k_{]}=q_{k}–q_{k}-1\text{for }K>k>1\\ \text{logit}\left(q_{k}\right)=c_{k}–risk\\ c_{k}∼\text{Dirichlet}\left(K\right) \end{array}$$

## Model definition

The primary outcome was a binary response: “If any family member was bitten by a dog, which approach would they adopt—traditional remedies or PEP?” Responses were coded as “1” for traditional remedies and “0” for PEP. We constructed two joint probabilistic models using Stan version 2.26.1 in the R Studio environment to understand factors associated with seeking care following a dog bite across dog-owning households [32]. Our first model was constructed assuming the observed vector of seeking care following a dog bite was drawn from a Bernoulli distribution with unknown parameters *p* (the unknown probability of seeking care). We modelled *p* using a logit link function and a linear predictor to estimate the additive effects of covariates on the probability of seeking care. A total of 9 parameters were estimated, including the intercept and a vector of individual sociodemographic factors, such as age, gender, education level, occupation, a latent variable to account for overall risk assessment, rural versus urban residence, access to veterinary care, poverty level, and road access. We accounted for the effect of potential regional sociocultural factors that might influence the probability of seeking care, as demonstrated by the findings from a recent study [33].

logit (*p*_*ij*_) = $\log\left(\frac{pij}{1-pij}\right)$ = β0 + β1*Age_*i*_ + β2* Gender_*i*_ + β3*Education_*i*_ + β4*Occupation _*i*_ + β5*latent risk _*i*_ + β6* Residence _*i*_ + β7*VetAccess _*i*_ + β8*Poverty _*i*_ + β9*RoadAccess _*i*_

Where *pij* is the probability that an individual *i* in region *j* seeks traditional care, *β0* the intercept of the model, β1, β1,…, β9 the fixed effects of each covariate (we used normal priors for the regression coefficient Betas).

In the second model, we added a random effect of locations where the survey was conducted to account for unobserved variation at the group level. This approach allowed us to capture variability across the states where the study was conducted, including regional influences such as religious practices that could affect the probability of seeking care. We used a normal prior for the random effect of regions, with mean 0 and a standard deviation sigma_region (standard deviation for random effects) ^u^region ∼N (0, ^σ^region).

A Cauchy prior with location 0 and scale 2.5 for the standard deviation of the region random effects was used: ^σ^region ∼Cauchy (0, 2.5), to allow for flexibility in estimating the degree of variability among regions where the study was conducted. We used Markov Chain Monte Carlo (MCMC) sampling to fit our models through Hamiltonian Monte Carlo (HMC) sampling [34, 35]. The sampler was configured to run 4,000 iterations for each chain, with four independent MCMC chains employed to ensure convergence. We checked the fit of our model to the data by visually examining diagnostic trace plots for the stability of simulation results. We computed R-hat values to confirm that all parameters had converged satisfactorily. The diagnostic checks indicated that Model 1 (without the spatial random effects) had suboptimal convergence, as evidenced by elevated R-hats and erratic, non-stationary behaviors in the trace plots. We relied on Model 2 for inference. Also, we generated summaries of posterior draws to obtain the model’s point estimates and credible intervals for all parameters. We classify regression coefficients as ‘significant’ if their 95% posterior credible intervals did not include 0.

## Results

### Descriptive statistics

Table 1 summarizes the details of the respondents who participated in this study.. Overall, 4,162 respondents participated in this study, with 27%, 95% Confidence Interval([Cl)] 26-27) indicating that they would seek traditional remedies in the event of a dog bite involving themselves or a family member.

**Table 1:** Preferences for allopathic vs. traditional intervention after dog bite among respondents by demographic, socioeconomic, and awareness factors.

Characteristics	*N* = 4,162	Traditional (%), n=1,112(%)	Allopathic (%), n=3,050
**Age**			
≥16	4,022	1,071 (96.3)	2,951(96.8)
< 16	140	41 (3.7)	99(3.2)
**Gender**			
Male	2,292	597 (53.7)	1,695 (55.6)
Female	1,870	515 (46.3)	1,355 (44.4)
**Occupation**			
Student	1,275	421 (37.9)	854 (67.0)
Civil Servant	779	99 (8.9)	680 (87.3)
Private/NGOs	2,108	592 (53.2)	1,516 (71.9)
**Level Of Education**			
None	554	174 (15.6)	380 (12.5)
Primary/Secondary	1,980	650 (58.5)	1,330 (43.6)
Tertiary	1,628	288 (25.9)	1,340 (43.9)
**Region Of Country**			
Kaduna	1,246	260 (23.4)	986 (32.3)
Anambra/Enugu	437	66 (5.9)	371 (12.2)
FCT	2,479	786 (70.7)	1,693 (55.5)
**Type Of Settlements**			
Rural Area	2,330	801 (72)	1529 (50.1)
Urban Area	1,832	311 (28)	1521 (49.9)
**Is there any livestock officer or Veterinary establishment in your location?**			
No/I Don’t Know	1,920	638 (57.4)	1,282 (42)
Available	2,242	474 (42.6)	1,768 (58)
**Have you heard about rabies?**			
Yes	1,112	468 (37.2)	644 (22.2)
No	3,050	789 (62.8)	2,261 (77.8)
**Bites from an infected animal cannot spread rabies to other animals?**			
True	1355	1041(34.1)	314(28.2)
False	2807	2009(65.9)	798(71.8)
**Rabies can infect the following?**			
I Don’t Know	806	307 (27.6)	499 (16.4)
Animal/Human	1,173	337 (30.3)	836 (27.4)
Both Animal and Human	2,183	468 (42.1)	1,715 (56.2)
**To your knowledge, have you or any members of your household been bitten by a dog in the last two years?**			
I Don’t Know	850	267 (24)	583 (19.1)
No	2,761	668 (60)	2,093 (68.6)
Yes	551	177 (16)	374 (12.3)

### Factors associated with the probability of seeking care following a dog bite

**Fig 2:**
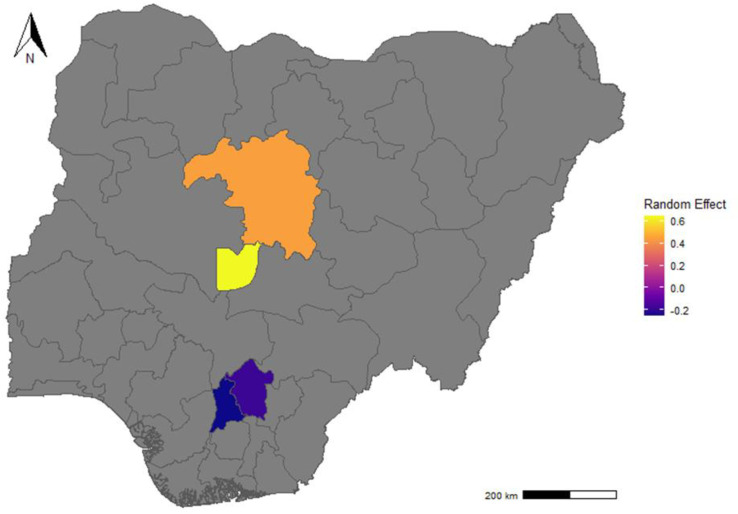
Geographical distribution of estimated random effects for the likelihood of Using traditional remedies across Nigeria, with more likelihood in the northern part of the country. The map was created using **R** (R Core Team, 2024), and the shapefile was retrieved from DIVA-GIS (https://www.diva-gis.org/).

**Fig 3:**
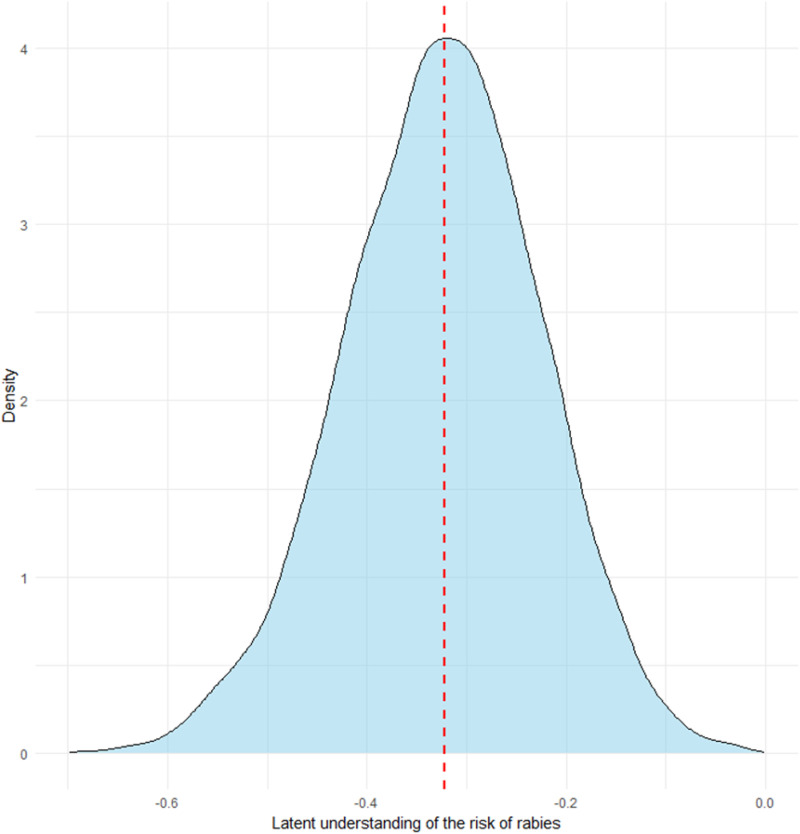
Posterior distribution of the effect of the latent variable on the probability of seeking traditional remedies after a potential dog bite indicates a decreased likelihood of individuals seeking traditional remedies.

We identified significant sociodemographic, socioeconomic, and infrastructural correlations with the use of traditional remedies. Our model results demonstrated a significantly higher likelihood of traditional remedy usage in northern than in southern regions of Nigeria (Fig 2). The latent variable was positively correlated with responses demonstrating a good understanding of rabies and negatively with responses inconsistent with an understanding of rabies (S1 Appendix). Male respondents were 24% more likely than female respondents to seek traditional remedies (odds ratio [OR]: 1.24; 95% CrI): 1.07-1.31), similarly individuals residing in rural areas reported 55% higher odds of using traditional remedies than those in urban areas (OR: 1.55; 95% CrI: 1.43–1.67). Respondents residing in areas with no veterinary services reported 35% higher odds of using traditional remedies than those near such facilities (OR: 1.35; 95% CrI: 1.15–1.42). Children under 16 years reported 27% lower odds of using traditional remedies than adults (OR: 0.73; 95% CI: 0.49–0.84). Private or unemployed individuals were more likely to use traditional remedies than civil servants (OR: 1.99; 95% Crl: 1.53-2.37). Also, respondents with tertiary education reported 42% lower odds of using traditional remedies than those without formal education (OR: 0.58; 95% CrI: 0.49–0.62). Our latent variable representing understanding of rabies risk was negatively associated with the probability of seeking traditional remedies (OR: 0.67; 95% CrI: 0.54–0.73) (Fig 3). Lastly, poverty was negatively associated with the likelihood of seeking traditional remedies (OR: 0.78; 95% CrI: 0.66–0.82) (Table 2).

**Table 2:** Regression coefficients for predictors of the probability of seeking traditional remedies.

Predictors	β coefficient (95% CrI)	Odds ratio (95% CrI)
**Age**		
≥16	Reference	Reference
< 16	-0.32 (-0.71- -0.18)	0.73 (0.49, 0.84)
**Gender**		
Female	Reference	Reference
Male	0.22 (0.07-0.27)	1.24 (1.07-1.31)
**Occupation**		
Civil Servant	Reference	Reference
Student	0.85(0.61-1.11)	2.34(1.84-3.03)
Private/NGOs	0.69(0.46-0.93)	2.00(1.53-2.37)
**Level Of Education**		
None	Reference	Reference
Primary/Secondary	-0.27 (-0.50- -0.19)	0.76 (0.61−0.83)
Tertiary	-0.54 (-0.72- -0.48)	0.58 (0.49–0.62)
**Latent variable**		
Latent variable	-0.40 (-0.61- -0.32)	0.67 (0.54−0.73)
**Type Of Settlements**		
Urban Area	Reference	Reference
Rural Area	0.44 (0.36-0.51)	1.55 (1.43−1.67)
**Is there any livestock officer or Veterinary establishment in your location?**		
Yes	Reference	Reference
No/I Don’t Know	0.30 (0.14- 0.35)	1.35 (1.15–1.42)
Poverty	**-**0.25 (-0.41- -0.20)	0.78 (0.66−0.82)
Road	0.10 (0.01- 0.13)	1.11 (1.01−1.14)

CrI- credible interval; NGOs- non-governmental organisation

## Discussion

Our study was conducted across three Nigerian states and the FCT to provide insights into the factors influencing health-seeking behaviour after dog bites. We found that 27% of respondents would seek traditional remedies following exposure to a dog bite—a rate lower than the 84% observed in Ethiopia [36] but much higher than Bhutan [37]. This finding is consistent with reports from other regions of the world [38–41], highlighting a persistent reliance on ineffective traditional remedies. This underscores the need for increased public education on seeking prompt medical attention and PEP after dog bites to prevent rabies virus transmission. Despite being lower, this is concerning and of significant public health concern, given that evidence suggests these methods are ineffective in preventing rabies deaths in humans. There have been reports of rabies deaths after using traditional remedies in Nigeria [3,42] and other parts of the world [43]. Furthermore, the finding underscores the need to engage traditional healers as rabies surveillance informants in rabies control programs. Such engagement would allow for a true One Health approach that recognizes the contribution of local knowledge systems [44]. Collaborating with traditional healers could provide opportunities to educate them on the limitations of their methods in rabies management, which may significantly help prevent human rabies deaths among those who seek their assistance. Indeed, accommodating the perspectives of religious and traditional leaders has previously contributed to wild polio eradication efforts in Nigeria [45]. We proposed the involvement of these influential community figures. Public health initiatives can build trust and improve the acceptance of medical interventions, thereby enhancing the effectiveness of disease control programs. We found that respondents aged 17 years and above were more likely to use traditional approaches than younger respondents. We suggest that older individuals may have a stronger inclination towards traditional remedies, possibly due to deeply rooted cultural beliefs. In contrast, younger respondents might be more aware of the importance of seeking prompt medical attention, as rabies awareness campaigns—such as those during World Rabies Day or mass dog vaccination drives—often target younger age groups, giving them a better understanding of the risks. This is consistent with a report from Nepal that found younger respondents had a better understanding of rabies than older respondents [46]. Moreover, parents may be more cautious about ensuring their children receive proper medical care after a dog bite.

Also, we observed regional variation in traditional remedies following dog bites. Respondents in the FCT were more likely to resort to traditional approaches than those in Anambra and Enugu, where only a few respondents indicated they would seek such remedies. This variation might be associated with these states’ demographics and cultural practices. Northern traditions predominantly influence the FCT due to the movement of people, especially from the northern part of the country, to the FCT, the country’s capital. The north educationally is less advanced compared to the southern part of the country [47]. This part of the country is known to have higher rates of vaccine dropouts, hesitancy due to socio-cultural and religious beliefs and reliance on traditional healing practices compared to the south [48–51]. In Hausa communities, often medicinal plants are the first option for healing [49]. Understanding these regional differences is crucial for developing tailored public health interventions that respect local customs while promoting effective rabies prevention strategies.

Based on our model, we found that respondents in rural areas reported 58% higher odds of using traditional remedies than those in urban areas. This disparity may be attributed to differences in access to healthcare facilities, awareness levels about rabies prevention, and cultural practices between urban and rural populations [41]. These findings are consistent with our model’s results, which suggested that residing in a location with no access to veterinary services (used as a proxy for healthcare access) was significantly associated with seeking traditional remedies as their first line of treatment. Urban areas typically have better healthcare infrastructure and more exposure to public health campaigns, which can increase awareness and accessibility of PEP. In contrast, rural areas may have limited access to medical services and more reliance on traditional remedies due to cultural beliefs or lack of information. This is consistent with a result from Chad that indicated that respondents in rural areas were more likely to seek care from a traditional healer [41]. This disparity highlights the need for targeted interventions to improve awareness and accessibility of PEP in rural communities.

We found that education and a latent variable—estimated using observed variables measuring understanding of rabies risk and literacy levels—were negatively associated with the probability of seeking traditional remedies. This finding is consistent with a report from Chad that suggests respondents with no school education were more likely to seek care from a traditional healer after a bite [41]. Lower or no educational background is linked to living in rural areas and lower income, constituting a double geographical and economic risk for access to human health and veterinary services [41]. This highlights the crucial role of education and awareness about rabies risks in prompting individuals to seek appropriate care following exposure to a dog bite.

Contrary to previous reports, poverty was negatively associated with the probability of seeking traditional remedies [19,52]. This discrepancy may be due to the way we measured poverty. While other studies estimated wealth scores based on four variables: (1) ownership of household items, (2) type of toilet facilities, (3) source of drinking water, and (4) household wall construction [53, 54], we estimated poverty using poverty raster data. Although poverty was not directly measured in our study, a possible explanation for our findings was that public health campaigns targeting low-income communities may have raised awareness about the importance of PEP, encouraging its use over traditional methods. Moreover, low-income individuals may place more trust in government-provided health services, especially if they have had positive experiences.

The strength of our study is that it was conducted in three Nigerian states plus the FCT with a large sample size. The findings can be useful in program management. However, there are a few limitations worth noting. First, the cross-sectional nature of this study limits causality assessment and demands further longitudinal studies. Second, the study was limited to surveying only dog owners, which may restrict the generalizability of the findings. For children who relied on their parents, parental influence may have impacted their decision-making. Standardized questionnaires may not cover all aspects of rabies, such as prevention, transmission, symptoms, and treatment. This can result in an incomplete assessment of an individual’s overall understanding. Also, qualitative interviews exploring the perspectives of individuals who reported a dog bite in their family could have identified prevalent practices and the factors influencing their decisions. Therefore, future investigations should use a mixed-method approach. Social desirability bias may have influenced some self-reported behaviours, particularly those related to seeking care for dog bites. Finally, while dog bite victims often seek both traditional and allopathic care, our study focused solely on which of the two options respondents were most likely to choose as their first point of care following potential exposure to a dog bit

## Conclusions

In our study, more than one-fourth of study participants reported that they would seek traditional remedies for a dog bite. This is concerning since canine rabies is endemic in Nigeria. Also, risk factors for seeking traditional remedies were high among adult respondents, and those residing in rural areas and without access to veterinary services. Our findings provide valuable information regarding healthcare-seeking behaviours and underscore the need for strategies to improve public education, enhance access to medical care, and integrate traditional healers into rabies management programs.

## Supporting information

S1 Appendix Map of the study area and raster maps used for the analysis. (DOCX)
