# Supplementary material for: Traditional practices versus modern healthcare: Determinants of traditional medicine use after potential dog bites among dog-owning households in Nigeria
Source: PLoS Negl Trop Dis. 2025 Mar 17;19(3):e0012910. doi: 10.1371/journal.pntd.0012910 (PMC11957387; doi:10.1371/journal.pntd.0012910)
Supplement: S1 Appendix — (DOCX) [file pntd.0012910.s001.docx]

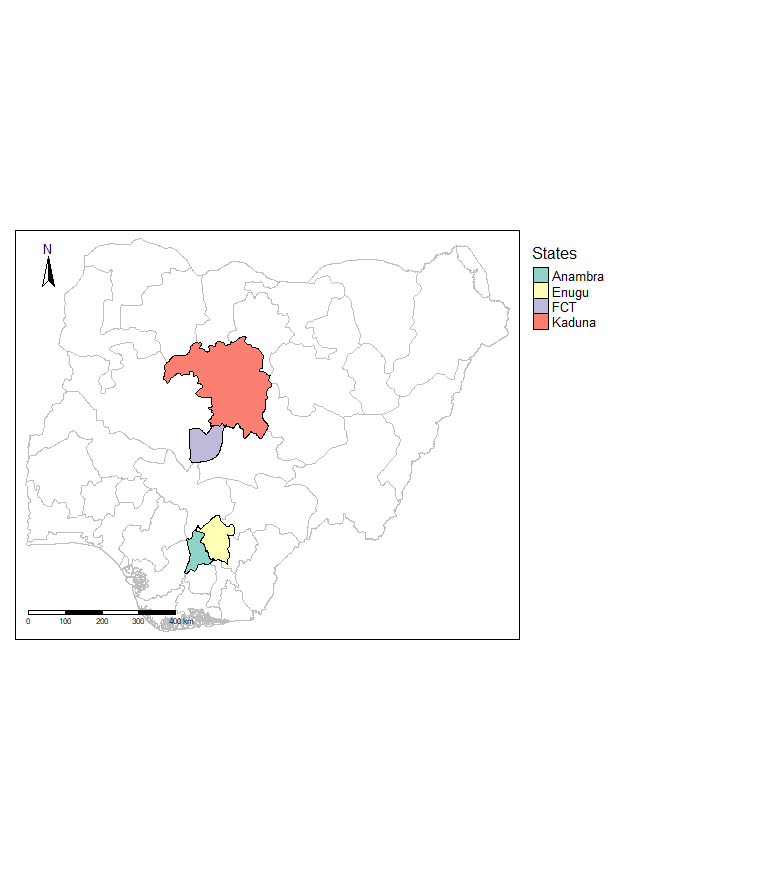


Fig A: The coloured areas on the map indicate the geographical locations within Nigeria where the study was conducted. These locations encompass Kaduna (Northwest), the FCT (North Central), and Anambra and Enugu (Southeast). The map was created using R [1], and the shapefile was retrieved from DIVA-GIS (https://diva-gis.org/).


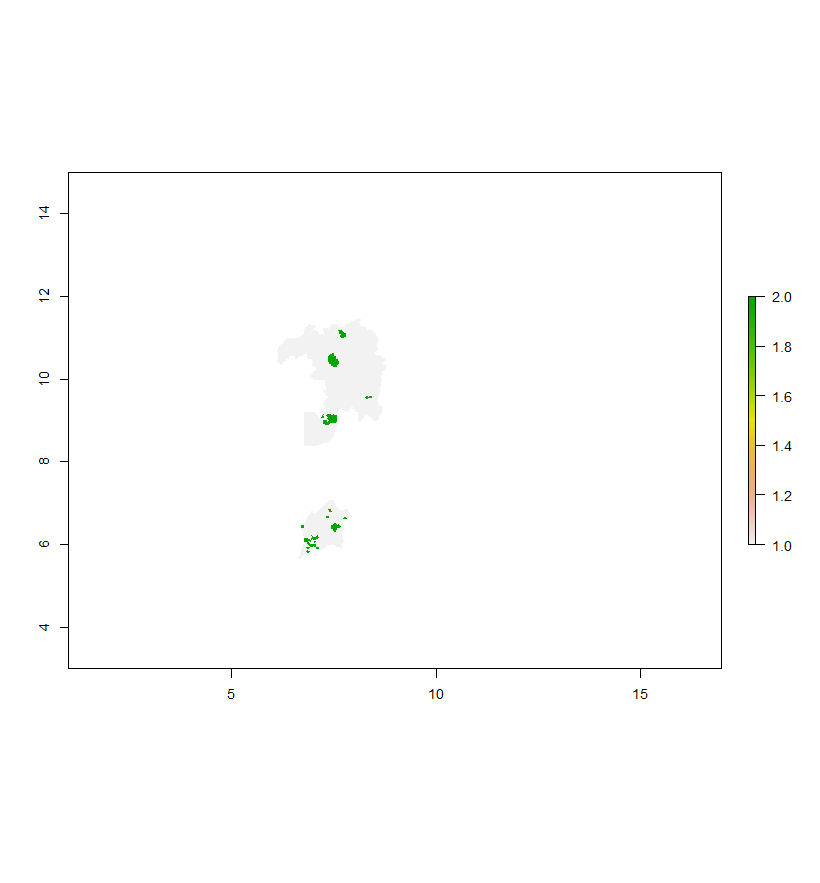


Fig B. Global Rural-Urban Mapping Project, Version 1 (GRUMPv1): Urban Extents Grid, VALUEInteger, where 1 = rural and 2 = urban[2]

The shapefile was retrieved from DIVA-GIS (https://diva-gis.org/).


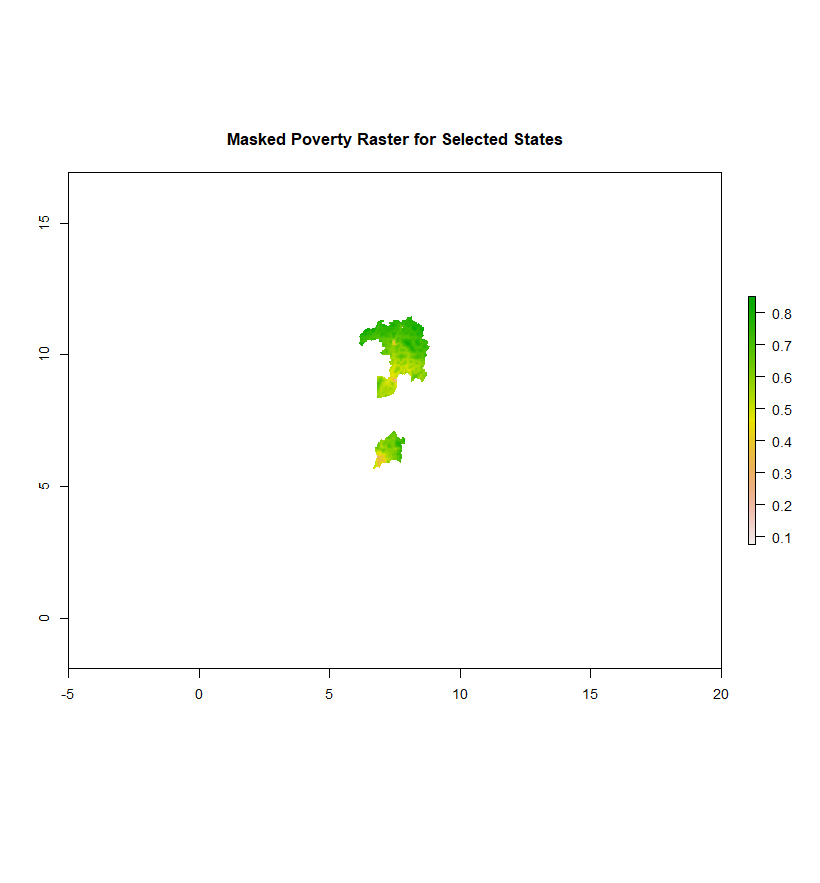


Fig C. Estimates of the proportion of people per grid square living in poverty, as defined by $1.25 a day and $2 a day thresholds and associated uncertainty metrics[3]. The map was created using R [1]. The shapefile was retrieved from DIVA-GIS (https://diva-gis.org/).


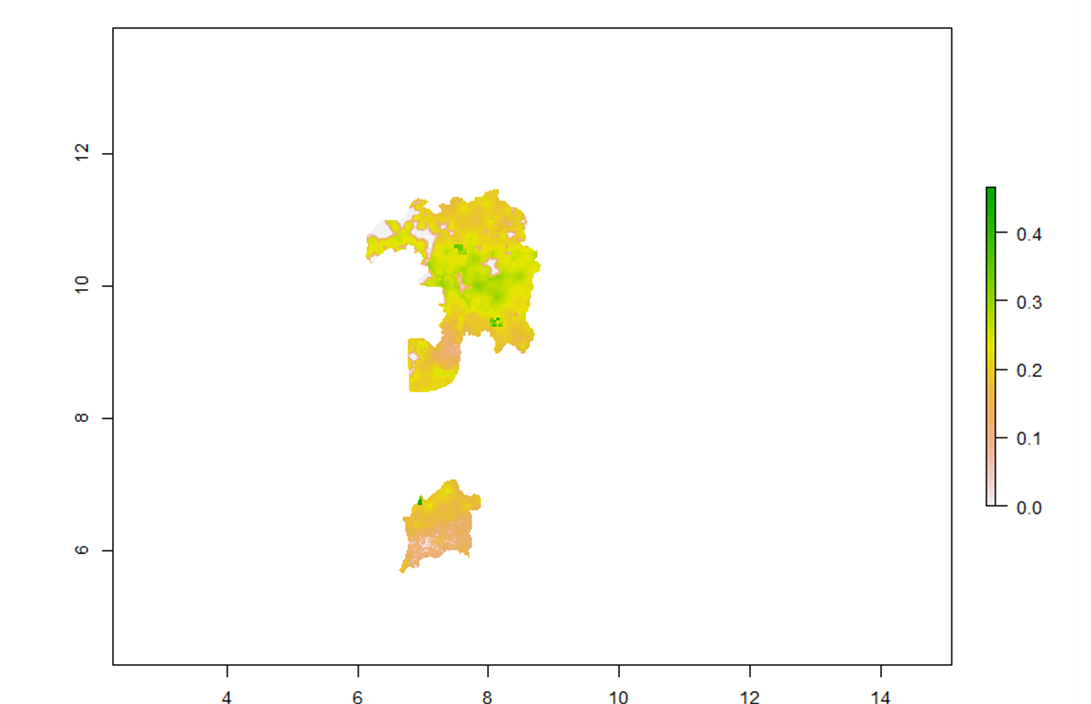


Fig D: Estimates of the proportion of men and women aged 15-49 per grid square that were classed as literate in 2013; the data series is comprised of four datasets, a) predicted proportion of (male/female) literacy (NGA_literacy_*.tif), and b) related uncertainty maps (NGA_literacy_*_interdecile.tif); note * = M or F (male/female)[4]. The shapefile was retrieved from DIVA-GIS (https://diva-gis.org/).


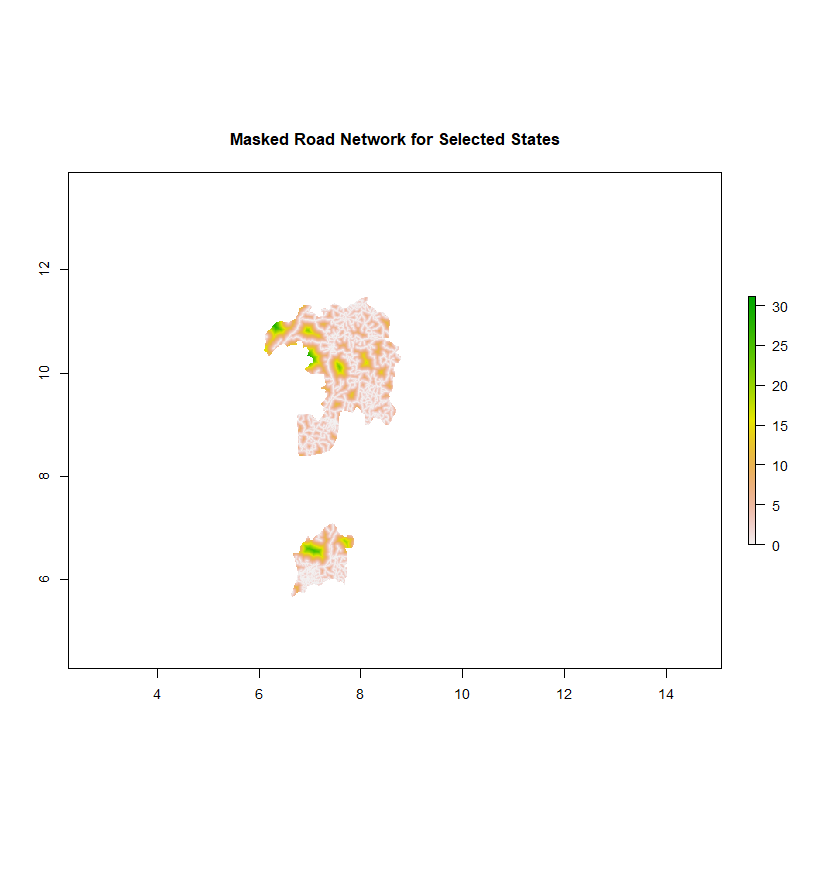


Fig E: Distance to road networks provides mean distances in meters from each grid cell:(https://data.humdata.org/ )

. #The projection is Geographic Coordinate System, WGS84. The values of the raster are the distance (in kilometres) #from the cell centre to the nearest feature. The map was created using R[3] . The shapefile was retrieved from DIVA-GIS (<https://www.diva-gis.org/>).

References

1. Team, R.C., *R: A language and environment for statistical computing. R Foundation for Statistical Computing, Vienna, Austria.* <http://www>. R-project. org/, 2016.

2. CIESIN, I., *The Global Rural-Urban Mapping Project, Version 1 (GRUMPv1): Urban Extents Grid.* Palisades, NY: NASA Socioeconomic Data and Applications Center (SEDAC). Accessed June, 2010.

3. Tatem, A., et al., *Pilot high resolution poverty maps, University of Southampton/Oxford*. 2013.

4. Bosco, C., et al., *Exploring the high-resolution mapping of gender-disaggregated development indicators.* Journal of The Royal Society Interface, 2017. **14**(129): p. 20160825.
